# Fish oil intake induces UCP1 upregulation in brown and white adipose tissue via the sympathetic nervous system

**DOI:** 10.1038/srep18013

**Published:** 2015-12-17

**Authors:** Minji Kim, Tsuyoshi Goto, Rina Yu, Kunitoshi Uchida, Makoto Tominaga, Yuriko Kano, Nobuyuki Takahashi, Teruo Kawada

**Affiliations:** 1 Laboratory of Molecular Function of Food, Division of Food Science and Biotechnology, Graduate School of Agriculture, Kyoto University, Uji, Kyoto 611-0011, Japan; 2 Research Unit for Physiological Chemistry, Kyoto University, Kyoto 606-8501, Japan; 3 Department of Food Science and Nutrition, University of Ulsan, Ulsan 680-749, South Korea; 4 Division of Cell Signaling, Okazaki Institute for Integrative Bioscience (National Institute for Physiological Sciences), National Institutes of Natural Sciences, Okazaki, Aichi 444-8787, Japan; 5 Department of Physiological Sciences, The Graduate University for Advanced Studies, Shonan Village, Hayama, Kanagawa 240-0193, Japan; 6 Laboratory of Nutrition Chemistry, Faculty of Home Economics, Kobe Women’s University, Kobe 654-8585, Japan

## Abstract

Brown adipose tissue (BAT) plays a central role in regulating energy homeostasis, and may provide novel strategies for the treatment of human obesity. BAT-mediated thermogenesis is regulated by mitochondrial uncoupling protein 1 (UCP1) in classical brown and ectopic beige adipocytes, and is controlled by sympathetic nervous system (SNS). Previous work indicated that fish oil intake reduces fat accumulation and induces UCP1 expression in BAT; however, the detailed mechanism of this effect remains unclear. In this study, we investigated the effect of fish oil on energy expenditure and the SNS. Fish oil intake increased oxygen consumption and rectal temperature, with concomitant upregulation of UCP1 and the β3 adrenergic receptor (β3AR), two markers of beige adipocytes, in the interscapular BAT and inguinal white adipose tissue (WAT). Additionally, fish oil intake increased the elimination of urinary catecholamines and the noradrenaline (NA) turnover rate in interscapular BAT and inguinal WAT. Furthermore, the effects of fish oil on SNS-mediated energy expenditure were abolished in transient receptor potential vanilloid 1 (TRPV1) knockout mice. In conclusion, fish oil intake can induce UCP1 expression in classical brown and beige adipocytes via the SNS, thereby attenuating fat accumulation and ameliorating lipid metabolism.

Fish oil, regarded as a healthy addition to the diet of diabetic patients^1^, contains the fatty acids eicosapentaenoic acid (EPA) and docosahexaenoic acid (DHA). These fatty acids, which are abundant in fish, have hypolipidaemic effects, augment the efficacy of the lipid-lowering drugs^2^, reduce cardiac events^3^, and inhibit the progression of atherosclerosis^4^. Numerous animal studies have demonstrated that fish oil reduces the accumulation of body fat, which could be mediated via several possible mechanisms, including reduced proliferation of fat cells^5^, and metabolic changes in the liver^6^, adipose tissue^7^, and small intestines^8^. Furthermore, fish oil supplementation prevents fat accumulation in white adipose tissue (WAT), compared to other dietary oils^9^.

Most mammals maintain body temperature by increasing heat production in response to cold. This process, termed adaptive thermogenesis, is achieved by shivering. Alternatively, humans and other small animals can increase energy expenditure via uncoupling oxidative phosphorylation in brown adipose tissue (BAT) of the interscapular region by BAT-specific uncoupling protein 1 (UCP1) in the mitochondria^10^,^11^,^12^. BAT-mediated thermogenesis is activated by the hypothalamus via the sympathetic nervous system (SNS). Increased noradrenaline (NA) release enhances cyclic adenosine monophosphate (cAMP) levels to rapidly activate lipolysis, thereby initiating mitochondrial heat production and synergistically increasing energy expenditure^13^,^14^,^15^. In contrast, WAT accumulates excess energy as triglycerides (TG), and primarily regulates energy storage.

Recent developments have demonstrated that brown adipocyte-like adipocytes, termed beige adipocytes, are found in the WAT, including the inguinal WAT of rodents and humans^16^,^17^,^18^,^19^,^20^. These adipocytes have a multilocular morphology and are UCP1-positive^21^. Furthermore, beige adipocytes are induced in response to cold exposure or prolonged β adrenergic stimulation^22^. Traditionally, WAT has been regarded as an organ for energy storage; however, a recent study indicated that activation of the SNS in WAT may regulate fat mobilization to maintain energy supplies^23^.

Dietary factors regulating brown and beige adipocyte development and function were identified in WAT. Many reports suggest that transient receptor potential vanilloid 1 (TRPV1) is activated by a wide range of chemical materials, including some lipids^24^,^25^,^26^. Recently, capsinoids, a group of capsaicin analogues, were shown to activate gastrointestinal TRPV1 and induce BAT thermogenesis in humans and rodents^27^. Furthermore, TRPV1 agonists found in foods, such as fish oil, could regulate TRPV1 in the gastrointestinal tract^28^,^29^.

In this study, we investigated the effect of fish oil intake on energy metabolism. Fish oil intake reduced body weight gain and fat accumulation, while increasing oxygen consumption and rectal temperature, as compared to control diet-fed mice. Furthermore, fish oil intake induced UCP1 expression in both of BAT and WAT, and activated the SNS. Combined, our data indicate that fish oil intake enhances energy utilization by inducing UCP1 in both BAT and WAT, and could thereby prevent obesity and related metabolic disorders.

## Results

### Effects of fish oil on body weight

**Figure 1:**
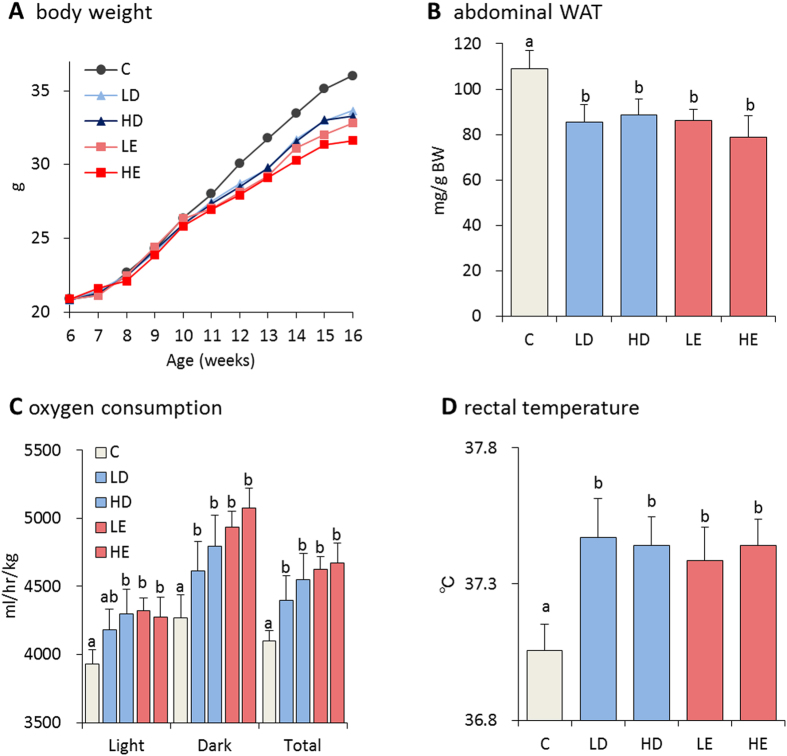
Fish oil intake reduced body weight gain and fat accumulation. Fish oil intake decreased body weight (**A**) and abdominal WAT (**B**) gain in mice. Mice were fed a control diet (**C**), control diet containing low-dose DHA-enriched fish oil (LD), control diet containing high-dose DHA-enriched fish oil (HD), control diet containing low-dose EPA-enriched fish oil (LE), and control diet containing high-dose EPA-enriched fish oil (HE) for 10 weeks. Fish oil intake increased oxygen consumption (**C**) and rectal temperature (**D**). Data represent the mean ± SEM of 7–8 animals per group. Different letters indicate significant differences between groups.

We evaluated the effect of fish oil on the accumulation of body fat in mice. Mice fed fish oil gained significantly less weight than mice fed a control diet (Fig. 1A). Furthermore, the abdominal adipose tissue weight was lower in fish oil treated mice than control mice (Fig. 1B and supplemental table 1). These data suggested fish oil intake increased energy expenditure. Fasting glucose, insulin, TG and leptin levels in the mice fed fish oil were lower than mice fed the control diet (Supplemental table 2). Additionally, the plasma adiponectin concentration was significantly higher in mice fed fish oil (Supplemental table 2). Taken together, fish oil intake decreased body weight and abdominal tissue weight, and protected against diet-induced obesity.

### Effect of fish oil on energy expenditure

To investigate how fish oil affected energy expenditure, indirect calorimetry was performed by measure oxygen consumption and carbon dioxide production. Oxygen consumption was significantly increased by fish oil intake, as compared to control diet intake, over a 20 h period (Fig. 1C). There was no statistically significant difference in respiratory quotient (RQ) between all groups (C; 0.86 ± 0.02, LD; 0.86 ± 0.02, HD; 0.88 ± 0.01, LE; 0.88 ± 0.01, HE; 0.88 ± 0.02). Furthermore, rectal temperature was significantly increased by fish oil intake (Fig. 1D). Consistent with the higher oxygen consumption and rectal temperature, the mice fed fish oil displayed enhanced energy expenditure. These results indicate that fish oil intake enhanced thermogenesis.

### Effect of fish oil on thermogenesis-associated gene expression

**Figure 2:**
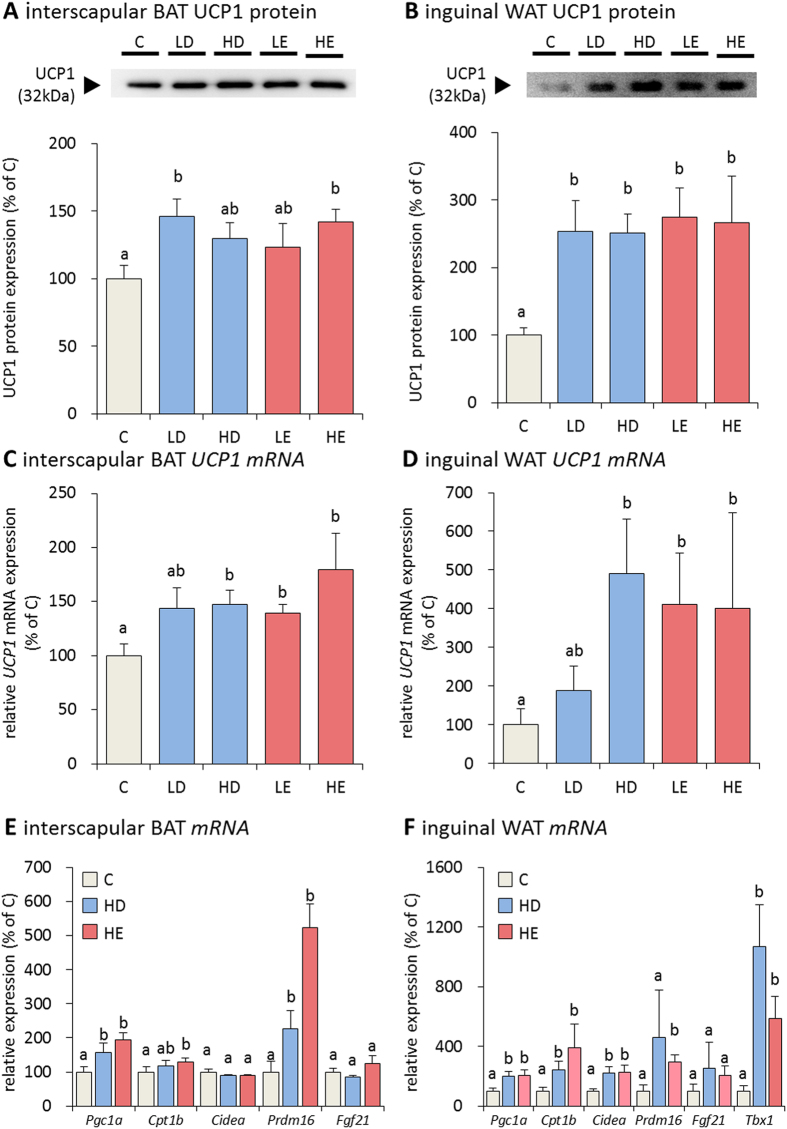
Fish oil intake induced UCP1 expression in interscapular BAT and inguinal WAT. Fish oil intake induced UCP1 protein expression in interscapular BAT (**A**) and inguinal WAT (**B**). Fish oil intake also induced *UCP1* mRNA expression in interscapular BAT (**C**) and inguinal WAT (**D**). Fish oil intake induced beige adipocyte-specific gene expression in interscapular BAT (**E**) and inguinal WAT (**F**). Data represent the mean ± SEM of 7–8 animals per group. Different letters indicate significant differences between groups.

We next examined the effects of fish oil on BAT gene and protein expression. UCP1 mRNA and protein expression in interscapular BAT was significantly induced by fish oil intake (Fig. 2A,C). Moreover, UCP1 mRNA and protein expression was also enhanced in the inguinal WAT (Fig. 2B,D). These UCP1-positive adipocytes were considered to be beige adipocytes. In addition, the expression of beige adipocyte marker genes, including peroxisome proliferator-activated receptor γ coactivator 1 α (*Pgc1*α), carnitine palmitoyltransferase 1 B (*Cpt1b*), cell death-inducing DFFA-like effector A (*Cidea*), PR domain containing 16 (*Prdm16*), and fibroblast growth factor 21 (*Fgf21*), was enhanced in interscapular BAT and inguinal WAT of mice fed fish oil (Fig. 2E). Interestingly, T-box transcription factor 1 (*Tbx1*), recently defined as a marker of beige adipocytes, was significantly induced in inguinal WAT by fish oil (Fig. 2F).

### Effect of fish oil on the SNS

**Figure 3:**
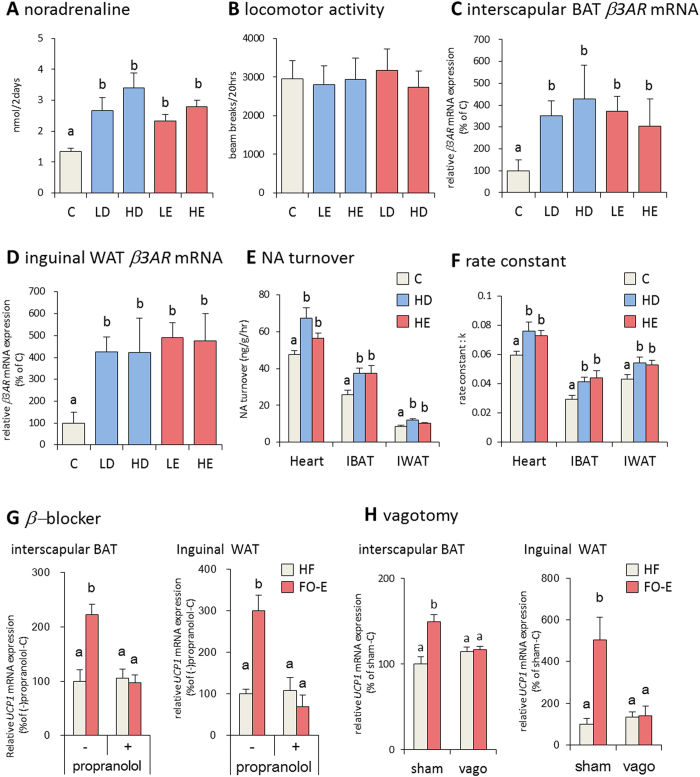
Fish oil intake induced SNS activation. Fish oil intake increased the amount of noradrenaline (**A**) in the urine. Urine was collected form mice housed in metabolic cages as described in the methods. Fish oil intake does not change total locomotor activity (**B**), after 20 h. Fish oil intake induced β*3AR* mRNA expression in interscapular BAT (**C**) and inguinal WAT (**D**). Effects of fish oil intake on NA turnover (**E**) and rate constant (**F**) in the heart, interscapular BAT, and inguinal WAT. Effects of propranolol on fish oil intake-induced changes of UCP1 expression levels in interscapular BAT(**G** *left*) and inguinal WAT (**G** *right*). Effects of fish oil on UCP1 expression levels in sham-operated (sham) and vagotomized (sham) mice (**H** *left*; interscapular BAT, (**H**) *right*; inguinal WAT). Mice were fed a control diet, and received oral administration of Mineral oil (HF) or EPA-enriched fish oil (FO-E). Data represent the mean ± SEM of 7–8 animals per group. Different letters indicate significant differences between groups.

BAT activity is primarily regulated by the SNS, through the binding of NA to the β adrenergic receptors, thereby inducing lipolysis and the activation of UCP1 to enhance thermogenesis. Thus, we next examined the effects of fish oil intake on the release of catecholamines in mice. The amount of NA in urine was significantly increased by fish oil intake (Fig. 3A). Additionally, we recorded spontaneous locomotor activity, and found that it was not altered by fish oil (Fig. 3B). The β3 adrenergic receptor (β3AR) activation contributes to thermoregulation and energy homeostasis via sympathetic stimulation of BAT adaptive thermogenesis. Therefore, we evaluated the effects of fish oil on β3AR expression. β3AR mRNA was significantly induced in interscapular BAT and inguinal WAT of mice fed fish oil, as compared to mice fed the control diet (Fig. 3C,D), and the histological analysis revealed clusters of multiocular cells with UCP1 expression (Supplemental Fig. 1). Furthermore, fish oil intake significantly enhanced the turnover of NA and rate constant in interscalupar BAT and inguinal WAT (Fig. 3E,F), suggesting that fish oil intake increased sympathetic outflow.

The increased expression of β3AR, UCP1, and beige adipocyte marker genes suggested that fish oil intake caused adipose tissues to activate the SNS. To determine the direct influence of fish oil activation in the SNS, we examined the effects of fish oil intake on the β adrenergic blocker, propranolol. After treatment with propranolol, fish oil intake did not affect upregulation of UCP1 mRNA expression in interscapular BAT and inguinal WAT (Fig. 3G). Furthermore, upregulation of UCP1 mRNA expression by fish oil intake was blocked by subdiaphragmatic vagotomy (Fig. 3H).

### Effect of fish oil on energy expenditure in TRPV1 knockout mice

**Figure 4:**
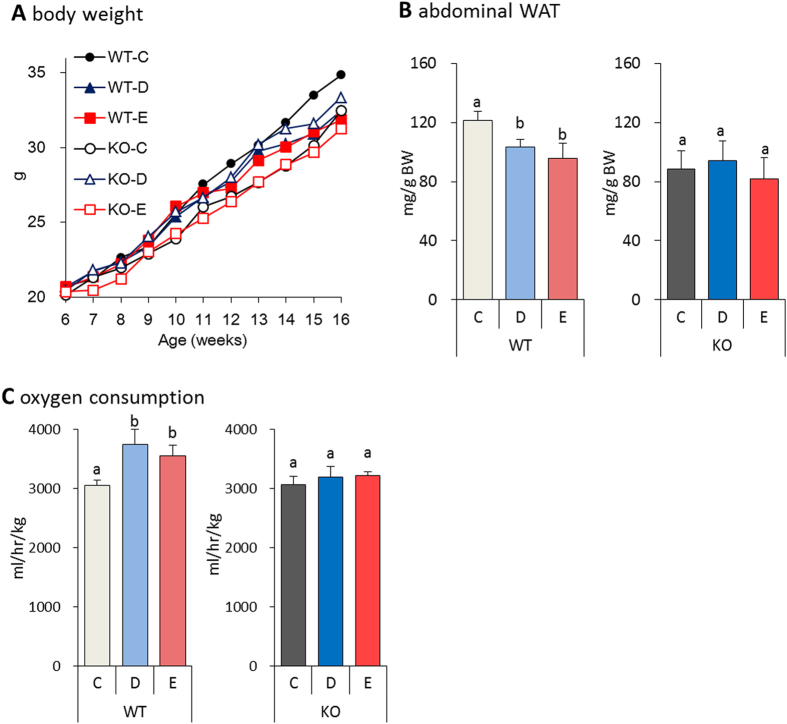
Fish oil intake reduced the body weight gain and fat accumulation in WT mice, but not TRPV1 KO mice. Fish oil intake decreased body weight (**A**) and abdominal WAT (**B**) gain in WT mice fed a control diet containing high-dose DHA-enriched fish oil (WT-D) and control diet containing high-dose EPA-enriched fish oil (WT-E), as compared to the control diet (WT-C) These effects were not observed in TRPV1 KO mice fed a control diet (KO-C), control diet containing high-dose DHA-enriched fish oil (KO-D), and control diet containing high-dose EPA-enriched fish oil (KO-E) for 10 weeks. Fish oil intake did not increase oxygen consumption (**C**) in TRPV1 KO mice. Data represent the mean ± SEM of 7–8 animals per group. Different letters indicate significant differences between groups.

According to previous studies, capsaicin and capsinoids, increase energy expenditure and reduce body fat, suggesting that TRPV1 may play a role in thermogenesis. Interestingly, dietary factors can also activate TRPV1. Stimulation of TRPV1 is known to activate the SNS^30^,^31^. However, how TRPV1 activates the SNS remains unclear. To better understand the role of TRPV1, we explored the effects of fish oil intake in wild-type (WT) mice and TRPV1 knockout (KO) mice. We examined changes in body weight of WT and TRPV1 KO mice consuming either a control diet or fish oil diet. In the TRPV1 KO mice fed fish oil, no difference in body weight gain and abdominal WAT accumulation was observed, as compared to TRPV1 KO mice fed the control diet (Fig. 4A,B). Interestingly, fish oil increased oxygen consumption in WT mice, but had no effect in TRPV1 KO mice (Fig. 4C). We also observed that WT mice fed fish oil showed lower fasting plasma glucose and TG concentrations compared to WT mice fed control diet (Supplemental table 3). No differences in fasting glucose and TG concentration were observed in the TRPV1 KO mice.

### Effect of fish oil on UCP1 expression in TRPV1 KO mice

**Figure 5:**
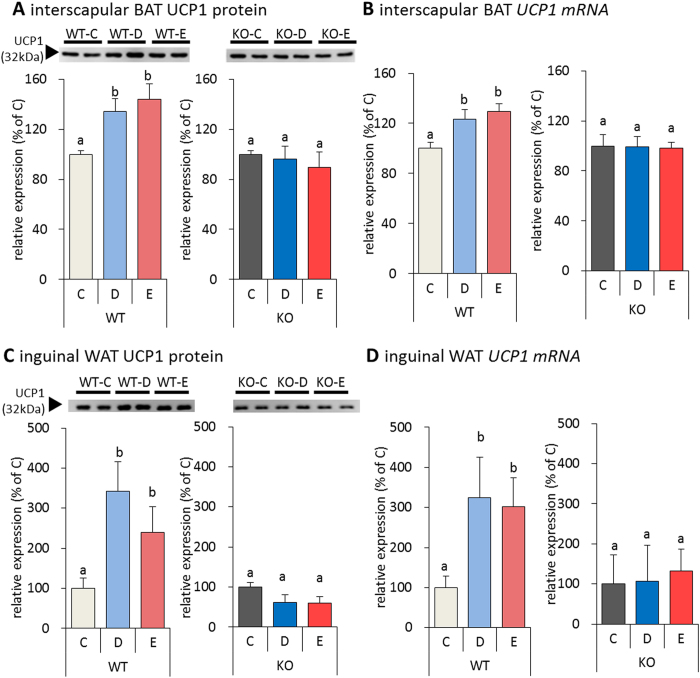
Fish oil intake did not induce UCP1 expression in TRPV1 KO mice. Fish oil intake did not enhance the UCP1 protein and mRNA expression levels in TRPV1 KO mice, as compared with WT mice, in the interscapular BAT (**A**,**C**) and inguinal WAT (**B**,**D**). Data represent the mean ± SEM of 7–8 animals per group. Different letters indicate significant differences between groups.

We next evaluated the effects of fish oil on the expression of UCP1 mRNA and protein in TRPV1 KO mice. The UCP1 mRNA and protein expression in the interscapular BAT was significantly induced in the fish oil fed WT mice, as compared to that the WT mice fed control diet. In contrast, TRPV1 KO mice fed either the fish oil or control diets were remarkably similar to WT mice fed a control diet (Fig. 5A,B). Moreover, the inguinal WAT of TRPV1 KO mice showed no changes in UCP1 mRNA and protein expression after fish oil treatment (Fig. 5C,D).

### Effect of fish oil on the SNS in TRPV1 KO mice

**Figure 6:**
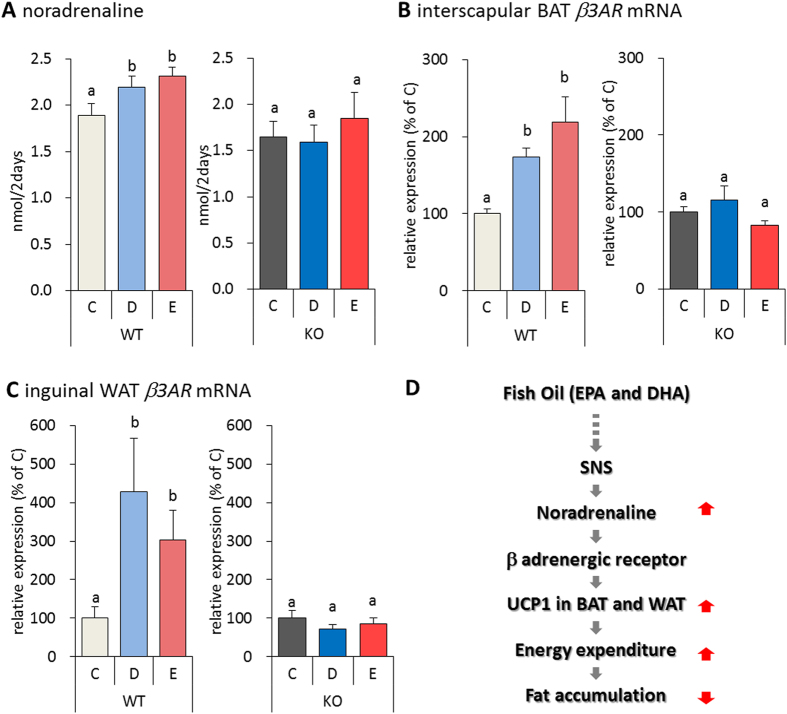
Fish oil intake did not induce SNS activation in TRPV1 KO mice. Fish oil intake increased the amount of urinary NA (**A**) in WT mice, but not in TRPV1 KO mice. Fish oil intake induced β*3AR* mRNA expression in the interscapular BAT (**B**) and inguinal WAT (**C**) of WT mice, but not TRPV1 KO mice. Data represent the mean ± SEM of 7–8 animals per group. Different letters indicate significant differences between groups. A schematic model of the effect of fish oil intake (**D**).

Finally, we evaluated the effect of fish oil intake on the SNS. As compared to WT mice, fish oil did not alter NA release in the urine of TRPV1 KO mice (Fig. 6A). Furthermore, upregulation of β3AR mRNA in interscapular BAT and inguinal WAT was not significantly altered in TRPV1 KO mice fed fish oil (Fig. 6B,C). Interestingly, fish oil intake significantly increased UCP1 induced by β adrenergic receptor in both interscapular BAT and inguinal WAT of WT mice; however, it had no effect on UCP1 and β3AR expression in TRPV1 KO mice.

## Discussion

In this study, we investigated the effects of fish oil intake on energy expenditure induced by UCP1. Consistent with previous studies, fish oil intake prevented the development of obesity; however, the mechanisms underlying the possible induction of thermogenesis and decreased fat accumulation remained unclear^32^,^33^. As shown previously in mice, the anti-obesity effect of fish oil could be dependent on lipid metabolism in the liver^34^, and fatty acid oxidation in the intestines^35^. Furthermore, as shown in this study, fish oil intake enhances oxygen consumption and rectal temperature. Thus, fish oil intake decreases in body weight gain and fat accumulation by increasing energy expenditure, suggesting that fish oil intake enhances thermogenesis.

UCP1-mediated thermogenesis in BAT plays an important role in the regulation of energy expenditure. Furthermore, UCP1 is a major determinant of BAT thermogenic activity^36^. Our data indicate that UCP1 expression in interscapular BAT (classical brown adipocytes) and inguinal WAT (beige/brite adipocytes) was increased by fish oil intake. Interscapular BAT and inguinal WAT share a number of BAT specific genes, such as *UCP1, Pgc1*α*, Cpt1b, Cidea, Prdm16*, and *Fgf21*; however, the two types of adipose tissue express these mRNAs at different levels. Interestingly, the inguinal WAT of fish oil-fed mice expressed beige adipocyte specific genes, such as *Tbx1*^37^. Furthermore, human BAT isolated from multiple locations, including the supraclavicular and retroperitoneal regions, abundantly express beige adipocyte-specific genes, indicating that human BAT is similar to beige adipocytes^38^. Recently, it was revealed that inducible beige adipocytes have potent thermogenic activity that is comparable to classical brown adipocytes^39^. Furthermore, immunochemical analysis of UCP1 reveals the presence of UCP1-positive multilocular adipocytes, a sign of beige adipocytes, in inguinal WAT after fish oil intake. Taken together, fish oil intake induced thermogenesis in both interscapular BAT and inguinal WAT.

Interscapular BAT is heavily innervated by the SNS, and NA released from the activated sympathetic nerves promotes thermogenesis by activating the β3AR^40^. Moreover, β3AR regulates the thermogenic functions of both brown and white adipocytes^40^. Fish oil intake increased catecholamine levels in the urine. In addition, fish oil intake increased NA turnover and rate constant in interscapular BAT and inguinal WAT. In particular, enhanced NA turnover and rate constant are considered a direct indicator of sympathetic activity in organs under sympathetic control^41^. The NA released from the SNS stimulates β3AR in interscapular BAT and inguinal WAT. Stimulation of β3AR leads to the induction of UCP1. Interestingly, β adrenergic blocker-treated and vagotomized mice showed the enhancement of UCP1 expression induced by EPA-enriched fish oil was canceled, suggesting that afferent vagal nerve in gastrointestinal tract mediates the stimulatory actions of fish oil. Taken together, our data indicate that fish oil intake can induce UCP1 in adipose tissues via the SNS.

TRPV4, a member of the TRPV family, is abundant in adipocytes and adipose tissues^42^,^43^. Ye *et al.* reported that mice that were intraperitoneally administered TRPV1 and TRPV4 antagonists showed increased browning (thermogenesis) of adipose tissues and were protected from diet-induced obesity^43^. However, our study suggested that dietary fish oil intake stimulated thermogenesis through the activation of TRPV1. This difference might have been a result of the different functions of TRPVs in different organs. Our additional experiments showed that both subdiaphragmatic vagotomy surgeries and treatment with a β-adrenergic blocker prevented the increase in UCP1 expression that was induced by the oral administration of fish oil. Moreover, TRPV1 expression in the gastrointestinal tract has been shown to play an important role in the activation of the sympathetic nervous system that is induced by capsinoids, which are TRPV1 agonists. Therefore, we speculated that the expression of TRPV1 in the gastrointestinal tract also plays an important role in the activation of the sympathetic nervous system that results from the enhancement of UCP1 expression that was induced by fish oil intake. However, Ye *et al.* reported that intraperitoneal injections of TRPV1 and TRPV4 antagonists directly inhibited Ca^2 + ^influx in adipocytes, which resulted in the induction of adipocyte browning^43^. These results indicated that organs in which TRPV1 is activated are important for the browning of adipocytes.

Dietary factors regulate the development and function of brown and beige adipocytes. Capsaicin in chili peppers, has been shown to enhance catecholamine secretion from the adrenal medulla through activation of the SNS^44^. Recently, capsinoids, a group of capsaicin analogues, were shown to activate gastrointestinal TRPV1 and induce BAT thermogenesis in humans^45^ and rodents^27^. Capsinoids-induced thermogenic sympathetic responses in BAT seem to require the activation of extrinsic nerves connected to the gastrointestinal tract^27^,^31^. Moreover, TRPV1 expressing afferent nerves were observed within gastrointestinal tracts^46^. However, TRPV1 has been reported to express in adipocytes^47^ and suggested to play roles in the regulation of energy metabolism^47^,^48^. Thus, we could not rule out the possibility of the contribution of TRPV1 to the fish oil-induced UCP1 expression in adipose tissues. Further studies are needed to clarify critical TRPV1-expressing tissues in which contribute to this phenomenon. Taken together, fish oil containing EPA and DHA, might activate SNS via the activation of TRPV1 expressing on the afferent nerves in the gastrointestinal tract, leading to the upregulation of UCP1 expression in interscapular BAT and inguinal WAT.

Body weight gain and fat accumulation were not decreased after fish oil intake in TRPV1 KO mice. Furthermore, fish oil intake did not increase oxygen consumption in TRPV1 KO mice. Additionally, UCP1 expression was significantly increased in WT mice following fish oil treatment, but not in TRPV1 KO mice. These findings indicate that TRPV1 plays an important role for upregulation of energy expenditure in response to fish oil intake. Interestingly, EPA and DHA can modulate TRPV1 activity directly and indirectly^29^. DHA and EPA displace TRPV1 ligand binding and evoke TRPV1 currents^28^. On the other hand, activation of TRPV1 by EPA and DHA requires activation of protein kinase C (PKC)^28^. PKC-dependent phosphorylation is known to increase the sensitivity of TRPV1^49^,^50^. In addition, thermal and chemical stimuli have been reported to activate TRPV1 synergistically^51^,^52^,^53^, and this synergistic activation was also suggested in the case of EPA and DHA^29^. Thus, it can be postulated that fish oil containing EPA and DHA may have a potential for activating TRPV1 through both direct and indirect manners. Taken together, fatty acids such as EPA and DHA or food ingredients, which activate TRPV1, may elicit sympathetic nerve activation, leading to UCP1-dependent thermogenesis in both interscapular BAT and inguinal WAT.

Alternatively, UCP1 upregulation by fish oil may arise via its anti-inflammatory activity. Indeed, EPA and DHA suppress obesity-induced adipose inflammatory responses by reduction of inflammatory cytokines production in co-cultured adipocytes/macrophage^54^,^55^, which is associated with anti-inflammatory macrophage M2 phenotype switching^55^. More importantly, we previously demonstrated that macrophage-mediated inflammatory cytokine such as tumor necrosis factor α suppresses the induction of UCP1 expression in white adipocytes via extracellular signal-regulated kinase activation in obese and diabetic conditions^56^. Hence, fish oil-induced upregulation of UCP1 in our observation may be at least in part attributed to its anti-inflammatory action in inguinal WAT.

In summary, fish oil activates and recruits interscapular BAT and inguinal WAT via activation of TRPV1, thereby increasing energy expenditure and decreasing body weight gain and fat accumulation. Based on these results, we propose that the SNS and BAT mediate the thermogenic effect of fish oil. Furthermore, fish oil-mediated thermogenesis via the SNS enhanced energy expenditure and reduced fat accumulation. A schematic model of this unique mechanism is shown in Fig. 6D. Thus, fish oil intake can induce UCP1 expression in classical brown and beige adipocytes via the SNS and TRPV1, and may contribute to an effective treatment for obesity.

## Materials and Methods

### Materials

DHA-enriched fish oil (DHA 25%, EPA 8%) was a gift from NOF Corporation (Tokyo, Japan) and EPA-enriched fish oil (EPA 28%, DHA 12%) was a gift from Nippon Suisan Kaisya, Ltd., (Tokyo, Japan). Noradrenaline (NA) and a-methyl-DL-tyrosine methyl ester hydrochloride (AMPT) were purchased from Sigma (St. Louis, MO, USA). Potassium dihydrogenphosphate, 3, 4-dihydroxybenzylamine hydrobromide (DHBA), and sodium 1-octanesulphonate were purchased from Nacalai Tesque (Kyoto, Japan).

### Experimental design and diets

C57BL/6 male mice were purchased form LSG Corporation (Tokyo, Japan). TRPV1-deficient (designated KO) C57BL/6 mice were generated by Caterina *et al.*^57^. All mice were housed separately at 23 ± 1 °C, maintained on a 12 h light/dark cycle, and fed a standard laboratory diet, MF (Oriental Yeast, Tokyo, Japan) for 1 week to stabilize the metabolic conditions before starting the experiments. The mice were divided into five groups (n = 7) according to the type of diet. The composition of diet, expressed as the percent of total calories, was 45% fat, 14% protein, and 41% carbohydrate with a caloric value of 4.74 kcal/g (Supplemental table 4). The concentration of fish oil on a diet with low-dose or high-dose was 1.2% and 2.4%, respectively. The energy intake of all the mice was equalized by pair feeding. All experiments were performed in accordance with the relevant guidelines and regulations of Kyoto University. And the animal care procedures and methods were approved by the Animal Care Committee of Kyoto University (Approved number: 26–49).

### Analysis of plasma TG, glucose, and insulin levels

The mice were fasted 5 h before blood collection. The plasma concentrations of glucose, TG, and insulin were determined by the Glucose C-test WAKO (Wako Pure Chemicals, Osaka, Japan), Triacylglycerol E-test Wako (Wako Pure Chemicals), and Morigana Ultrasensitive Mouse Insulin Assay Kit (Morinaga Institute of Biological Science, Yokohama, Japan), respectively. All kits were used according to manufacturer’s instructions, with the same blood samples.

### Measurement of oxygen consumption, RQ and locomotor activity

The oxygen consumption and RQ were measured using an indirect calorimetric system (Oxymax Equal Flow 8 Chamber/Small Subject System; Columbus Instruments, Columbus, OH, USA) equipped with an eight-chamber airtight metabolic cage^58^. Mice were acclimated to the individual metabolic cages for 2 hours prior to the experiment. The data for each metabolic cage were collected every 9 min, with room air as a reference, and measured for 20 h. The RQ was calculated by dividing the CO_2_ production by the O_2_ consumption, and was used to estimate the contribution of fats and carbohydrates to *in vivo* whole-body energy metabolism in mice^59^. The locomotor activity was measured using an Actimo-S (Bio Research Centre, Nagoya, Japan).

### RNA preparation and quantification of gene expression

Total RNA was prepared from animal tissues by using Sepasol-RNA I Super reagent (Nacalai Tesque) in accordance with manufacturer’s protocol. Total RNA was revers-transcribed using M-MLV reverse transcriptase (Promega Corporation, Fitchburg, WI, USA) in accordance with manufacturer’s instructions using a thermal cycler (Takara PR Thermal Cycler SP, Takara Bio Inc., Shiga, Japan). To quantify mRNA expression, real-time PCR was performed with a Light cycler system (Roche Diagnostics, Mannheim, Germany) by using SYBR Green fluorescence signals^53^. Expression data were normalized to mouse *36B4*.

### Mitochondrial preparations

Interscapular BAT and inguinal WAT mitochondria were prepared as described by Cannon and Lindberg^60^. The tissues were minced with scissors and homogenized in 300 mM sucrose solution with protease inhibitors. The homogenates were centrifuges at 8,500 × g for 10 min at 4 °C. After removing the fat layer and supernatant, the pellets were resuspended, and the nuclei and cell debris were removed by centrifugation at 800 × g for 10 min. The resulting supernatants were centrifuged at 8,500 × g for 10 min. The final pellets containing the crude mitochondrial fraction were resuspended in a small volume of 300 mM sucrose solution.

### Western blotting

The protein concentration was determined by using a protein assay kit (Bio-Rad laboratories, Hercules, CA, USA). Proteins were diluted with Laemmli SDS-PAGE sample buffer and bolied for 5 min in the presence of 2-mercaptoethanol. The samples underwent sodium dodecyl sulphate-polyacrylamide gel electrophoresis (SDS-PAGE) using a 12.5% gel, followed by transfer to an Amersham Hybond-LFP polyvinylidene fluoride (PVDF) membrane (GE Healthcare, Little Chalfont, UK). The membranes were blocked with 5% skimmed milk and 0.1% tween-20 in PBS for 1 h, as previously described^61^. After blocking, the membranes were incubated in a rabbit anti-UCP1 antibody (Sigma) overnight at 4 °C. Next, the membranes were incubated in HRP-conjugated anti-rabbit IgG (Santa Cruz Biotechnology, San Antonio, TX, USA) for 2 h at room temperature. Protein bands were visualized by ELC chemiluminescence detection (Millipore, Bedford, MA, USA). Immunoreactive protein bands were quantified by using the Image J software (NIH, Bethesda, MD, USA).

### Histological analysis

The interscapular BAT, and inguinal WAT, were fixed in 4% paraformaldehyde and embedded in paraffin. For UCP1 immunohistochemistry, paraffin-embedded sections (6μm) were incubated with anti-UCP1 (Sigma), followed by detection using the ABC^62^ method. Nuclei were counterstained with modified Mayer’s hematoxylin (Merck, Darmstadt, Germany)^62^.

### Extraction of tissue catecholamines

The heart, interscapular BAT, and inguinal WAT were rapidly removed, and frozen in liquid nitrogen. DHBA was added as an internal standard, and the organs were homogenized in 1 mL of 0.4 M perchloric acid (PCA). After centrifugation, the catecholamines in the supernatant were purified with activated alumina, as described previously^41^.

### Extraction of urine catecholamines

Mice were acclimated to the individual metabolic cages for 2 hours prior to the experiment, and urine samples were collected for 48 h. The urine was collected in 1 mL of 6 M HCl solution for collection and storage. Urine samples were purified via activated alumina, as previously described^63^.

### Catecholamine assays

Catecholamines were eluted with 100 μL of 0.4 M PCA. Catecholamines were assayed by high-performance liquid chromatography (HPLC) with electrochemical detection^63^. The detector potential was set at 700 mV maintained across a glassy carbon working electrode. Methanol-buffer (10:90, v/v) composed of 50 mM potassium phosphate buffer (pH 3.5), 10 μM EDTA·2Na, and 100 mg/L sodium 1-octanesulphonate was used as the mobile phase at flow rate of 1 mL/min.

### Measurement of NA turnover rate

The experiment was started at 8 AM and measured by determining the concentration of NA in the heart, interscapular BAT, and inguinal WAT at 0, 8, and 16 h following intraperitoneal injection of AMPT (250 mg/kg). The organs were rapidly dissected, weighed, and frozen in nitrogen to measure NA content. The NA turnover rate was calculated as the slope of the decline in log-trans-formed NA concentration after intraperitoneal injection of AMPT^64^.

### Treatment with propranolol

Mice were given an intraperitoneal injection of propranolol (2mg/kg)^65^. Thirty minutes after the injection, mice were given an oral administration of fish oil. After four hours, mice were decapitated and the organs were rapidly dissected, weighed, and frozen in nitrogen.

### Subdiaphragmatic vagotomy

Mice were anesthetized with pentobarbital sodium (15 mg/kg ip). After laparotomy, the two trunks of the vagus nerve were identified under an operating microscope, silk sutures were tied a few millimeters apart around each vagal trunk before bifurcation of the gastric branch and the hepatic and celiac branches, and the nerve was cut between the sutures^66^. For the sham operations, the vagus was similarly exposed but neither ligated nor cut. All operated mice lost weight for the 2–3 days after surgery, and the mortality rate reached 15%. On the basis of changes in body weight, ingestion patters became normal 3 days after vagotomy.

### Statistical analysis

The data are presented as the mean ± standard error of mean (SEM). All statistical analyses were performed using SPSS 12.0 for Windows (SPSS Inc. Armonk, NY, USA). Statistical differences between the experimental groups were assessed by ANOVA, followed by the Turkey-Kramer HSD *post hoc* test. Different letters indicate significant differences between groups (p < 0.05).

## Additional Information

**How to cite this article**: Kim, M. *et al.* Fish oil intake induces UCP1 upregulation in brown and white adipose tissue via the sympathetic nervous system. *Sci. Rep.* **5**, 18013; doi: 10.1038/srep18013 (2015).

## Supplementary Material

Supplementary Information
