# Supplementary material for: Fish oil intake induces UCP1 upregulation in brown and white adipose tissue via the sympathetic nervous system
Source: Sci Rep. 2015 Dec 17;5:18013. doi: 10.1038/srep18013 (PMC4682086; doi:10.1038/srep18013)
Supplement: Supplementary Information [file srep18013-s1.pdf]

Fish oil intake induces UCP1 upregulation in brown and white adipose tissue via the sympathetic nervous system

Minji Kim[1], Tsuyoshi Goto[1,2], Rina Yu[3], Kunitoshi Uchida[4,5], Makoto Tominaga[4,5], Yuriko Kano[6], Nobuyuki Takahashi[1,2], Teruo Kawada[1,2,*]

[1] Laboratory of Molecular Function of Food, Division of Food Science and Biotechnology, Graduate School of Agriculture, Kyoto University, Uji, Kyoto 611-0011, Japan
[2] Research Unit for Physiological Chemistry, Kyoto University, Kyoto 606-8501, Japan
[3] Department of Food Science and Nutrition, University of Ulsan, Ulsan 680-749, South Korea
[4] Division of Cell Signaling, Okazaki Institute for Integrative Bioscience (National Institute for Physiological Sciences), National Institutes of Natural Sciences, Okazaki, Aichi 444-8787, Japan
[5] Department of Physiological Sciences, The Graduate University for Advanced Studies, Shonan Village, Hayama, Kanagawa 240-0193, Japan
[6] Laboratory of Nutrition Chemistry, Faculty of Home Economics, Kobe Women`s University, Kobe 654-8585, Japan

[*]Corresponding author: Teruo Kawada FAX: +81-774-38-3752; Email: fat@kais.kyoto-u.ac.jp

Supplemental table 1. Organ weights in 16-week-old male C57BL mice

mg/gBW	C	LD	HD	LE	HE
**Inguinal WAT**	24.7±0.7^{a}	22.8±1.2^{ab}	22.1±1.5^{b}	20.3±1.0^{b}	19.3±1.6^{b}
**Epididymal WAT**	48.2±2.6^{ab}	34.5±2.8^{b}	37.4±3.5^{b}	37.7±3.6^{b}	35.0±2.9^{b}
**Mesenteric WAT**	15.4±1.0^{a}	11.6±1.0^{b}	12.8±1.0^{b}	15.5±1.3^{ab}	9.9±1.8^{b}
**Renal WAT**	20.9±0.8^{a}	16.5±0.8^{b}	16.3±1.2^{b}	12.0±0.6^{b}	14.7±1.4^{b}
**Interscapular BAT**	4.2±0.3^{a}	3.4±0.2^{b}	3.6±0.2^{b}	3.5±0.2^{b}	3.4±0.4^{b}
**Liver**	37.2±0.7^{a}	36.0±1.6^{a}	37.4±1.5^{a}	36.4±0.7^{a}	36.7±1.0^{a}
**Gastrocnemius**	11.1±0.5^{a}	11.0±0.5^{a}	11.0±0.7^{a}	12.0±0.6^{a}	11.8±0.5^{a}

Different letters were indicated significant differences among groups.

Supplemental table 2. Plasma concentrations of various parameters in 16-week-old male C57BL mice

	C	LD	HD	LE	HE
**TG (mg/dL)**	105.2±5.2[a]	75.5±3.9[b]	74.0±2.6[b]	80.9±6.4[b]	77.9±3.0[b]
**Adiponectin (μg/mL)**	7.0±0.4[a]	8.8±0.3[b]	9.4±0.5[b]	8.8±0.4[b]	9.3±0.5[b]
**Leptin (μg/mL)**	33.7±4.0[a]	24.5±1.9[b]	26.8±1.2[b]	25.6±1.2[b]	21.2±4.4[b]
**Glucose (mg/dL)**	222.4±8.1[a]	199.3±7.7[b]	192.3±7.2[b]	192.5±5.2[b]	178.3±9.6[b]
**Insulin (ng/mL)**	1.0±0.1[a]	0.5±0.1[b]	0.4±0.1[b]	0.4±0.1[b]	0.6±0.1[b]

Different letters were indicated significant differences among groups.

Supplemental table 3. Plasma concentrations of glucose and TG in 16-week-old male C57BL and TRPV1 KO mice

	WT-C	WT-D	WT-E
Glucose (mg/dL)	220.9±4.3[a]	195.9±7.7[b]	193.8±9.9[b]
TG (mg/dL)	74.4±2.0[a]	61.0±2.3[b]	63.5±2.7[b]

	KO-C	KO-D	KO-E
Glucose (mg/dL)	215.3±9.7[a]	222.1±15[a]	218.7±8.1[a]
TG (mg/dL)	63.0±4.1[a]	62.0±2.8[a]	59.8±6.1[a]

Different letters were indicated significant differences among groups.

Supplemental table 4. Composition of experimental diets

Ingredients (g/100 g)	C	LD	HD	LE	HE
Casein	15	15	15	15	15
L-cystine	0.4	0.4	0.4	0.4	0.4
Corn starch	10.9	10.9	10.9	10.9	10.9
Maltodextrin	11.7	11.7	11.7	11.7	11.7
Sucrose	25.8	25.8	25.8	25.8	25.8
Cellulose	5.9	5.9	5.9	5.9	5.9
Corn oil	2.9	2.9	2.9	2.9	2.9
Lard	20.9	19.7	18.5	19.7	18.5
Fish oil	-	1.2	2.4	1.2	2.4
Mineral mixture	5.3	5.3	5.3	5.3	5.3
Vitamin mixture	1.2	1.2	1.2	1.2	1.2

Supplemental figure 1. Representative images of immunohistochemistry for UCP1 in interscapular BAT (A) and inguinal WAT (B). Left, Hematoxylin and eosin staining. Right, immunostaining with UCP1 antibody. Origin magnification, X200.

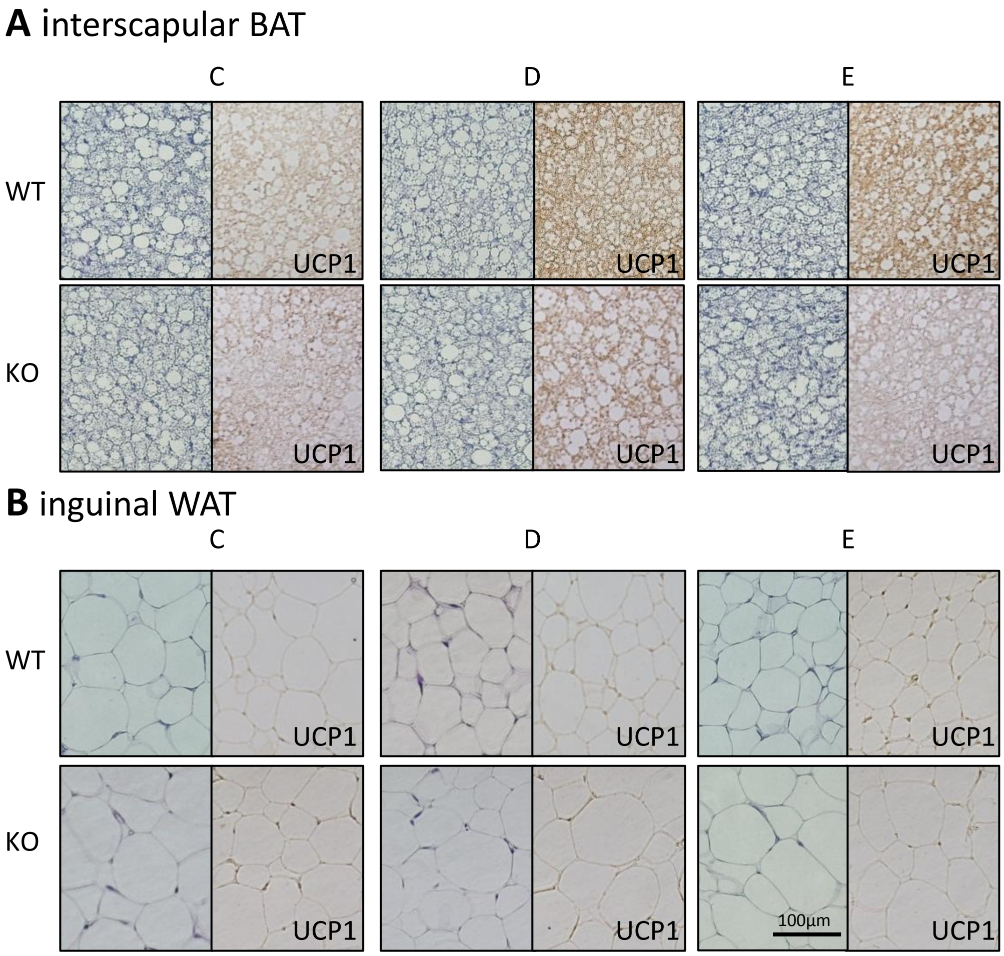

Supplemental figure 1 Kim MJ *et al*
